# Reliability and validity of the elements of desire questionnaire in premenopausal women with hypoactive sexual desire disorder

**DOI:** 10.1186/s41687-020-00241-6

**Published:** 2020-10-08

**Authors:** Dennis A. Revicki, Stanley E. Althof, Leonard R. Derogatis, Sheryl A. Kingsberg, Hilary Wilson, Amama Sadiq, Julie Krop, Robert Jordan, Johna Lucas

**Affiliations:** 1 Revicki Outcomes Research Consulting, 5656 Eastwind Drive, Sarasota, FL 34233 USA; 2 Case Western Reserve University School of Medicine, Cleveland, OH USA; 3 Maryland Center for Sexual Health, Lutherville, MD USA; 4 University Hospitals Cleveland Medical Center, Cleveland, OH USA; 5 Evidera, Bethesda, MD USA; 6 AMAG Pharmaceuticals, Inc., Waltham, MA USA; 7 Palatin Technologies, Inc., Cranbury, NJ USA

**Keywords:** Bremelanotide, Female sexual dysfunction, Hypoactive sexual desire disorder, Patient-reported outcomes, Validation

## Abstract

**Background:**

The Elements of Desire Questionnaire (EDQ) is a patient-reported outcome (PRO) measure developed to evaluate sexual desire and was included in two identically designed phase 3 clinical trials (RECONNECT) as an exploratory endpoint. The EDQ was developed based on a literature review, qualitative research with patients with hypoactive sexual desire disorder (HSDD), and input from clinical experts. This instrument is intended to be used to collect efficacy data in clinical trials evaluating potential treatments for HSDD. The objective of this study was to evaluate the measurement properties of both the monthly and daily recall versions of the EDQ during the RECONNECT trials.

**Methods:**

Participants completed the EDQ daily version for 7 consecutive days prior to selected monthly clinic visits. The monthly recall version was completed at each monthly clinic visit. The analysis population consisted of all subjects with Female Sexual Function Index (FSFI) data at baseline and ≥ 1 follow-up visit.

**Results:**

At baseline, 1144 and 676 subjects completed the monthly and daily recall EDQs, respectively. The EDQ scores had good internal consistency and test-retest reliability. Monthly and daily recall EDQ scores were correlated with FSFI-desire domain scores at baseline and month 3. Scores from the monthly and daily recall versions were also correlated. After 6 months, there was a significantly greater improvement for bremelanotide versus placebo in both the monthly and daily recall versions (both *P* < 0.0001).

**Conclusions:**

The results demonstrated that EDQ exhibited good reliability, validity, and sensitivity to change. Consistent with other validated PRO measures of sexual desire, the EDQ provides additional insights into sexual desire.

**Trial registration:**

NCT02338960 and NCT02333071 (RECONNECT studies).

## Background

Hypoactive sexual desire disorder (HSDD) is defined as a lack of motivation for sexual activity or loss of desire to initiate or participate in sexual activity accompanied by significant personal distress [1]. HSDD is the most common form of female sexual dysfunction (FSD) in the United States, affecting 8.9% of women aged 18–44 years [2]. Women with HSDD reported several psychological consequences, including personal feelings of concern, unhappiness, hopelessness, anger, as well as loss of femininity and altered self-esteem [3]. However, evaluation of the condition has been complicated by the evolution of the clinical definition over the past 20–30 years [1, 4–6]. Indeed, the Diagnostic and Statistical Manual of Mental Disorders (DSM) definition of HSDD has evolved from a condition that applied to both men and women in DSM-IV to being included within “female sexual interest/arousal disorder” in DSM-5 [6, 7]. Due to the biopsychosocial complexity of HSDD and its impact on the quality of patients’ lives, psychometrically sound patient-reported outcome (PRO) instruments are required to assess the efficacy of potential treatments [8].

Flibanserin and bremelanotide are the only drugs approved by the US Food and Drug Administration (FDA) for the treatment of acquired, generalized HSDD in premenopausal women [9, 10]. These agents have demonstrated sustained efficacy as measured by change from baseline to end-of-study in the Female Sexual Function Index–desire domain (FSFI-D) and Female Sexual Distress Scale–Revised (FSDS-R) scores as co-primary endpoints, and have an acceptable safety profile in premenopausal women [11–19].

The FSFI-D has been used most frequently to assess desire and interest in clinical trials for HSDD. The desire scale consists of two items on frequency and level of sexual desire; however, some researchers and regulatory agencies have expressed concerns that these two items may not capture the complete experience of women’s sexual desire and interest.

The Elements of Desire Questionnaire (EDQ), a recently developed PRO measure to evaluate sexual desire, was included in the bremelanotide phase 3 RECONNECT trials as an exploratory endpoint. This instrument was developed to more comprehensively measure the attributes of desire that are not currently captured by the FSFI-D, such as intensity of sexual desire, thoughts/fantasies about sex, and receptivity to sexual requests, with an objective of measuring the effectiveness of drugs for this condition with regard to more facets of the pathology and in accordance with the FDA guidelines for the development of PRO measures to support labeling claims [20]. The EDQ is intended to be used in clinical trials evaluating potential treatments that may improve sexual desire in women with HSDD; both 24-h (daily) and 28-day (monthly) recall versions were developed. There is evidence supporting 7- to 28-day recall for assessing sexual desire, compared with daily assessment, as more consistent with women’s experience [21].

### Study objectives

The purpose of this study was to evaluate the measurement properties of both the monthly and daily recall versions of the EDQ, and to assess the efficacy of bremelanotide as measured by the EDQ in the context of the RECONNECT studies.

## Methods

### RECONNECT study design

The RECONNECT studies were two identically designed, randomized, double-blind, placebo-controlled trials (NCT02333071 [Study 301] and NCT02338960 [Study 302]) that evaluated the efficacy and safety of bremelanotide versus placebo in premenopausal women with HSDD either with or without decreased arousal [18]. Both multicenter studies, which were undertaken in the United States and Canada, comprised a single-blind 4-week placebo run-in treatment period, a 24-week double-blind treatment period (core study phase), and an optional 52-week open-label extension (OLE) phase [19]. The studies were conducted in accordance with Good Clinical Practice requirements, and all participants provided written informed consent. During the core study phase, participants self-administered bremelanotide 1.75 mg or placebo subcutaneously via autoinjector pen, on demand, prior to sexual activity. Bremelanotide significantly improved sexual desire and decreased related distress as measured by the co-primary endpoints, change from baseline to end-of-study in scores from the FSFI-D and Item 13 of the Female Sexual Distress Scale–Desire/Arousal/Orgasm (FSDS-DAO). The recently developed EDQ (described below) was included as an exploratory endpoint in the RECONNECT studies.

### Analysis population

All analyses were performed in the modified intent-to-treat (mITT) study population, which was defined as all subjects who received at least one dose of study drug during the double-blind treatment period and had at least one post-dose follow-up visit. The analysis population consisted of all subjects with FSFI data at baseline and at least one follow-up visit. Data from the FSFI, FSDS-DAO, and other PRO measures were used to evaluate the measurement properties of the EDQ.

### Elements of desire questionnaire (EDQ)

The EDQ is a 9-item PRO questionnaire intended to evaluate the efficacy of potential new treatments for women with HSDD (Table 1). The EDQ was developed based on a previous literature review, input from clinical experts, and qualitative research that was conducted to evaluate the content validity, and provides a broader range assessment of women’s experiences with low sexual desire and generates data around elements of desire such as intensity of sexual desire, thoughts/fantasies about sex, and receptivity to sexual requests, which are not present in existing scales [22]. There are monthly and daily recall versions of the EDQ. The daily recall version was developed to be consistent with FDA guidelines for PROs [20] and to evaulate the inherent issues with daily data collection (i.e., missing data and poor fit with the sexual desire construct). The EDQ monthly recall version was completed at each monthly clinic visit, and enables participants with infrequent sexual activity and lack of day-to-day variation in sexual desire to avoid the task of completing a daily diary. Participants also completed the EDQ daily version for 7 consecutive days prior to visits at screening (visit 1), baseline (visit 3), month 3 (visit 6), and month 6 (visit 9) during the core phase of the RECONNECT studies. Response options for questions are described in Table 1.

**Table 1:** EDQ: monthly and daily recall versions

**Monthly Recall Version**	
1. Over the past 4 weeks, how often did you feel sexual desire or interest?	
2. Over the past 4 weeks, what was the intensity of your sexual desire or interest?	
3. Over the past 4 weeks, how often did you have thoughts or fantasies about sexual activities?	
4. Over the past 4 weeks, how would you rate your interest in engaging in sexual activity?	
5. Over the past 4 weeks, how often did you want to have sexual activity?	
6. Over the past 4 weeks, how would you rate your receptivity to your partner’s sexual requests?	
7. Over the past 4 weeks, how often did you initiate a sexual activity?	
8. Over the past 4 weeks, how would you rate your interest in initiating sexual activity?	
9. Over the past 4 weeks, how would you rate your satisfaction with your level of desire or interest in sexual activity?	
**Daily Recall Version**	
1. Over the past 24 h, how often did you feel sexual desire or interest?	
2. Over the past 24 h, what was the intensity of your sexual desire or interest?	
3. Over the past 24 h, how often did you have thoughts or fantasies about sexual activities?	
4. Over the past 24 h, how would you rate your interest in engaging in sexual activity?	
5. Over the past 24 h, did you want to have sexual activity?	
6. Over the past 24 h, how would you rate your receptivity to your partner’s sexual requests?	
7. Over the past 24 h, did you initiate a sexual activity?	
8. Over the past 24 h, how would you rate your interest in initiating sexual activity?	
9. Over the past 24 h, how would you rate your satisfaction with your level of desire or interest in sexual activity?	

*EDQ* Elements of Desire Questionnaire

Question 1, 3, 5, and 7 response options: 1–Never; 2–Rarely; 3–Sometimes; 4–Often; 5–Always (for daily recall version questions 5 and 7, the specific response options are 1-Yes or 2-No). Question 2, 4, and 8 response options: 1–Absent; 2–Mild; 3–Moderate; 4–Strong; 5–Very Strong. Question 6 response options: 1–Absent; 2–Low; 3–Moderate; 4–High; 5–Very High; 0–Partner did not initiate. Question 9 response options: 1–Very dissatisfied; 2–Moderately dissatisfied; 3–Neither satisfied nor dissatisfied; 4–Moderately satisfied; 5–Very satisfied

The EDQ monthly recall score is derived by calculating a mean total score based on the sum of the nine EDQ items. For the daily recall version, a mean total score is calculated based on the mean daily score for Items 1, 2, 3, 4, 6, 8, and 9 for the 7-day period before a scheduled study visit. Items 5 and 7 are assessed using a binary response option (yes/no) and are not included in the EDQ weekly total score. A minimum of daily diary data from at least 4 days per week were required to calculate an EDQ weekly total score.

### Additional patient-reported outcome (PRO) instruments

#### Female sexual function index (FSFI)

The FSFI is a validated 19-item measure of female sexual function that consists of six domains: desire, arousal, lubrication, orgasm, satisfaction, and pain [23]. The FSFI total score consists of the sum of the domain scores and ranges from 2 to 36. Of note, the FSFI-D consists of two additional questions and the score range is from 1.2 to 6.0. It has a recall period of 4 weeks, with higher scores indicating greater level of sexual function.

#### Female sexual distress scale–desire/arousal/orgasm (FSDS-DAO)

The FSDS-DAO is a validated 15-item instrument based on the 13-item FSDS-R [24, 25]. Item 13 relates specifically to “bother” related to sexual desire and has a recall period of 4 weeks. For this instrument, higher scores indicate greater sexually related distress.

#### General assessment questionnaire (GAQ)

The GAQ consists of four items related to satisfaction level while using the study drug, including satisfaction with arousal, satisfaction with desire, degree of benefit while on study drug, and impact of taking the study drug on the relationship with their partner. Participants answer the questions on their overall satisfaction while using the study drug. Responses are selected on a 7-point numeric rating scale from 1 (very much worse) to 7 (very much better).

#### Female sexual encounter profile–revised (FSEP-R)

The FSEP-R is a 10-item measure designed to assess sexual encounters, including initiation, level of desire, satisfaction with arousal, lubrication, arousal, ability to achieve orgasm, and satisfaction with the sexual encounter [26]. Participants complete the FSEP-R within 24 h after each sexual encounter, whether or not study drug was used before that encounter. A “sexual encounter” is defined as any act involving sexual contact with genitalia and/or oral mucosa, and includes intercourse, oral sex, and masturbation by self or a partner.

#### Women’s inventory of treatment satisfaction (WITS-9)

The WITS-9 assesses satisfaction with treatment and sexual relations over the past 4 weeks [27]. Participants answer the nine items on a 7-point numeric rating scale from − 3 (very unsatisfied or very likely not to continue) to 3 (very satisfied or very likely to continue). The total score is calculated as the average of the scores from the nine questions and ranges from − 3.0 to 3.0; a higher score on the WITS-9 indicates a higher level of satisfaction with treatment.

### Analyses

#### Internal consistency reliability

The internal consistency reliability of the EDQ was evaluated by examining the item-item correlations for the EDQ using Cronbach’s formula for coefficient alpha (α) [28], which was calculated for the EDQ score(s) at baseline, month 3, and month 6 separately for the monthly and daily recall versions**.** Alpha coefficients between 0.7 and 0.9 indicate good internal consistency; between 0.4 and < 0.7 indicate moderate internal consistency; and < 0.4 indicate low internal consistency reliability [28, 29]. Cronbach’s α > 0.7 is generally considered acceptable for group comparisons [30].

#### Test-retest reliability

Test-retest reliability was assessed by an intra-class correlation coefficient (ICC) and paired *t*-tests of the monthly recall EDQ scores at screening (visit 1) and the start of the single-blind placebo period (visit 2). The period between visit 1 and visit 2 was a no-drug qualification period, and therefore subjects’ HSDD symptoms were expected to remain stable. For the daily diary version of the EDQ, test-retest was evaluated using the first single-day score and last single-day score available in the week leading up to study visit 2.

#### EDQ factor analysis

Factor analysis was conducted to examine the conceptual framework of the EDQ. We expected a single underlying factor given that the various desire-related concepts were strongly associated; however, a two-factor solution was also examined. The total sample was split into two random groups of approximately equal size: the first split sample was used to conduct an exploratory factor analysis (EFA), and the second to conduct a confirmatory factor analysis (CFA). Separate factor analysis models were run for both the 24-h and monthly recall versions of EDQ. The EFA included all nine items from the EDQ, with no pre-specified factors. Corresponding eigen-values were examined empirically to determine the number of factors [31]. Standardized root mean square (RMS) residual and RMS error of approximation were examined to assess model goodness-of-fit. Good model-fit is indicated when values of standardized RMS and RMS error of approximation are both < 0.08 [32–34]. Approximation of simple structure with factor loading ≥0.40 was the criterion for accepting a factor solution; oblique rotation was allowed. If an item loaded high on two or more factors, the item was flagged for deletion [35]. Following completion of EFA, a CFA model was conducted in the second split half sample to confirm the solution identified in the EFA. The fit of the CFA model was assessed with comparative fit index, RMS error of approximation, and weighted RMS residual. A comparative fit index > 0.90, RMS error of approximation < 0.07, and weighted RMS residual close to 1 was considered an acceptable fit [33, 34]. The CFA was conducted using Mplus software [35].

#### EDQ validity analysis

To examine the validity of the EDQ, the pattern and magnitude of the relationships with EDQ score(s) and other indicators of female sexual function were examined with Spearman’s correlations using data from baseline and month 3. Seven-day average scores were used for the EDQ daily diary analysis. Spearman’s correlations between the EDQ and the following measures were evaluated: FSFI-total, FSFI-D, FSDS-DAO total, FSDS-DAO Item 13, FSEP-R desire, GAQ-2 (desire), and WITS-9 total. No formal hypotheses were specified for the correlations between the EDQ and the other sexual function measures. However, we did specify the expected strength of these correlations a priori. Correlations were classified as small (≤0.3), moderate (0.3–0.6), or large (≥0.6) [36]. Spearman correlations were expected to be large between the EDQ score(s) and other desire-specific item and scale scores (FSFI-D, FSEP-R, and GAQ-2). Spearman correlations between the EDQ and other measures of female sexual function (e.g., orgasm and lubrication) were expected to be statistically significant, but smaller in magnitude compared to those with the desire-specific scores.

#### Known-groups validity

Known-groups validity is the extent to which scores from an instrument are distinguishable from groups of subjects that differ by a relevant clinical or other indicator. The validity was evaluated by comparing the mean EDQ scores at baseline, month 3, and month 6 in patients with varying degrees of HSDD severity, as defined by the FSDS-DAO (< 11 vs. ≥11), GAQ desire item (≥5 vs. < 5), and number of satisfying sexual events (SSEs) (< 2 vs. ≥2 events) [24, 37]. Severity groups were compared using Student’s *t*-tests to evaluate mean differences in FSFI scores at baseline, month 3, and month 6 for both the monthly recall and 24-h recall versions of the EDQ. The 7-day average EDQ scores were used for the 24-h recall version.

#### Relationship between the monthly and daily recall versions

The relationship between the monthly and daily recall versions of the EDQ scale scores was explored to inform the agreement between these two different recall periods. Spearman’s coefficients and ICCs were calculated between the monthly recall EDQ scores and the 7-day weekly average daily diary EDQ scores at baseline and month 3.

#### EDQ ability to detect changes

The ability to detect change was evaluated by comparing the mean change in EDQ scores from visit 2 (baseline) to visit 9 (month 6) among patients who had achieved the following definitions of response at visit 9: FSDS-DAO (< 11 responder vs. ≥11 nonresponder), GAQ desire item (≥5 responder vs. < 5 nonresponder), and number of SSEs (< 2 nonresponder events vs. ≥2 responder events). Responder groups were defined at month 6 based on the FSDS-DAO, GAQ desire item, and number of SSEs in the same manner as described above in the known-groups validity section.

### Statistical analysis

For the analysis evaluating the measurement properties of the EDQ, data were pooled across the two studies and treatment groups. All statistical tests were two-tailed Student’s *t*-tests and were conducted with type I error probability fixed at 0.05. The mean and standard deviations were described for continuous variables, and percent distributions by category were described for categorical variables.

For the efficacy analysis, data were pooled across studies but not across treatment groups. Wilcoxon rank-sum tests were used to compare change from baseline to end-of-study in EDQ total scores (monthly and daily recall versions) across treatment groups (bremelanotide vs. placebo). In addition, mixed-model repeated measures were conducted to evaluate change from baseline of all double-blind treatment period (core study phase) data in the EDQ total score.

## Results

### Participants and baseline characteristics

The analysis population consisted of 1147 subjects who were randomized to treatment, had a baseline (visit 3) FSFI score, and for whom at least one follow-up FSFI score was available. The mean age of participants in the RECONNECT studies was 38.7 years and mean body mass index (BMI) was 28.6 kg/m^2^. The majority of RECONNECT participants were white (85.6%) and were diagnosed with HSDD with decreased arousal (71.3%). At baseline, 1144 and 676 participants completed the monthly and daily recall EDQ, respectively.

### EDQ reliability

#### Internal consistency reliability

Both monthly and daily recall versions of the EDQ demonstrated high (α > 0.9) internal consistency throughout baseline to 6 months (Table 2). Cronbach’s α did not increase or decrease appreciably with any item removed.

**Table 2:** EDQ internal consistency: monthly and daily recall versions

EDQ Score	Baseline		3 Months		6 Months	
	N	Cronbach’s α	N	Cronbach’s α	N	Cronbach’s α
**Monthly Recall**	1144	0.924	966	0.952	836	0.956
With item removed:						
Q1. How often sexual desire or interest		0.912		0.945		0.949
Q2. Intensity of sexual desire or interest		0.911		0.944		0.948
Q3. Sexual thoughts or fantasies		0.917		0.948		0.952
Q4. Interest in engaging in sex		0.909		0.943		0.947
Q5. Want to have sex		0.911		0.943		0.948
Q6. Receptivity to partner’s request		0.924		0.952		0.954
Q7. You initiate sex		0.923		0.951		0.957
Q8. Interest in initiating sex		0.912		0.944		0.949
Q9. Satisfaction with desire or interest		0.919		0.948		0.953
**Single-Day** **Daily Recall**	924	0.949	837	0.963	700	0.969
With item removed:						
Q1. How often sexual desire or interest		0.938		0.956		0.963
Q2. Intensity of sexual desire or interest		0.939		0.955		0.961
Q3. Sexual thoughts or fantasies		0.943		0.957		0.964
Q4. Interest in engaging in sex		0.936		0.953		0.960
Q6. Receptivity to partner’s requests		0.936		0.958		0.963
Q8. Interest in initiating sex		0.939		0.955		0.962
Q9. Satisfaction with desire or interest		0.959		0.969		0.974

*EDQ* Elements of Desire Questionnaire

#### Test-retest reliability

Test-retest reliability was evaluated in all subjects within approximately 1 month, from screening (visit 1) to start of the single-blind placebo period (visit 2). There was no treatment intervention during this period, and therefore no expectation of a change in clinical condition. The monthly version of the EDQ had a greater ICC for test-retest reliability than the daily version (0.63 in the monthly recall version vs. 0.33 in the daily recall version) (Table 3). Of note, there was a substantially higher rate of missing data for the daily recall version relative to the monthly recall version of the questionnaire. The missing data correspond to infrequency of participants’ current sexual activity and the lack of day-to-day variations experienced with sexual desire as explanations for missing diary data. Notably, the ICC values improved when limited to a stable population, as defined by a change of no more than ±5% in the FSDS-DAO total score (monthly recall scores = 0.68; daily recall weekly scores = 0.58). ICC values > 0.70 are generally considered acceptable for establishing test-retest reliability [30].

**Table 3:** EDQ test-retest reliability

**EDQ Version**	**n**	**Visit 1,** **Mean (SD)**	**Visit 2,** **Mean (SD)**	**Difference Score**^**a**^	**T-value**	***P*-value**^**b**^	**ICC**
Monthly recall	1130	1.60 (0.41)	1.63 (0.42)	0.03 (0.35)	2.83	0.0048	0.63
**EDQ Version**	**n**	**Day 1/Visit 2, Mean (SD)**	**Day 7/Visit 2, Mean (SD)**	**Difference Score**^**a**^	**T-value**	***P*-value**^**b**^	**ICC**
Daily recall	848	1.46 (0.49)	1.45 (0.55)	−0.01 (0.60)	− 0.25	0.8062	0.33

*EDQ* Elements of Desire Questionnaire, *ICC* Intra-class correlation coefficient, *SD* Standard deviation

^a^Calculated as difference between respective time points

^b^Paired *t*-tests comparing responses at days 1 and 7

### EDQ validity

Validity was explored through examination of the pattern of correlations between the EDQ scores and the other PRO measures that were included in the clinical trials, including the total FSFI, FSFI-D, total FSDS-DAO, FSDS-DAO Item 13, GAQ Item 2, FSEP-R desire, and total WITS-9 scores. Results from baseline and month 3 for the monthly recall version are summarized in Table 4. Correlation coefficients between the EDQ and other measures of desire were moderate to large, providing support for the convergent validity of the EDQ: FSFI-D (baseline = 0.79; month 3 = 0.87); GAQ Item 2: satisfaction with desire (baseline = 0.52; month 3 = 0.66); and FSEP-R desire item (baseline = 0.60; month 3 = 0.64). Correlation coefficients between the EDQ and a measure of treatment satisfaction, the WITS-9 total, were also large (baseline = 0.65; month 3 = 0.76). As expected, the correlation coefficient between the EDQ and the more distally related concept of pain (FSFI) was small (baseline = 0.06; month 3 = 0.15). Results for the daily recall version were comparable to the monthly recall version, with moderate to large correlations between the EDQ score and alternate measures of desire, and smaller correlations with FSFI pain (Table 4). Based on prespecified expectations, 6 of 7 (86%) correlations at baseline and 7 of 7 (100%) correlations at month 3 were confirmed. Comparable correlations were observed for the EDQ daily version (Table 4). For the EDQ daily version, 5 of 7 (71%) correlations at baseline and 6 of 7 (86%) correlations at month 3 were confirmed based on prespecified expectations.

**Table 4:** EDQ correlation with the FSFI, FSDS, and other patient-reported outcome scores: monthly and daily recall versions

	Monthly Recall Version		Daily Recall Version	
	R	***P***	R	***P***
**Baseline**	*n* = 1142–1144		*n* = 674–676	
FSFI Total	0.63	< 0.0001	0.54	< 0.0001
FSFI-D	0.79	< 0.0001	0.70	< 0.0001
FSDS-DAO Total	−0.54	< 0.0001	−0.42	< 0.0001
FSDS-DAO Item 13	−0.51	< 0.0001	−0.40	< 0.0001
GAQ Item 2	0.52	< 0.0001	0.37	< 0.0001
FSEP-R desire	0.60	< 0.0001	0.57	< 0.0001
WITS-9 Total	0.65	< 0.0001	0.53	< 0.0001
**Month 3**	*n* = 963–967		*n* = 423–444	
FSFI Total	0.75	< 0.0001	0.68	< 0.0001
FSFI-D	0.87	< 0.0001	0.81	< 0.0001
FSDS-DAO Total	−0.61	< 0.0001	−0.55	< 0.0001
FSDS-DAO Item 13	−0.60	< 0.0001	−0.51	< 0.0001
GAQ Item 2	0.66	< 0.0001	0.59	< 0.0001
FSEP-R desire	0.64	< 0.0001	0.64	< 0.0001
WITS-9 Total	0.76	< 0.0001	0.69	< 0.0001

*EDQ* Elements of Desire Questionnaire, *FSDS-DAO* Female Sexual Distress Scale–Desire/Arousal/Orgasm, *FSEP-R* Female Sexual Encounter Profile–Revised, *FSFI* Female Sexual Function Index, *FSFI-D* Female Sexual Function Index–desire domain, *GAQ* General Assessment Questionnaire, *WITS-9* Women’s Inventory of Treatment Satisfaction (9 questions)

### Known-groups validity

Participants in the less severe groups based on each anchor variable (FSDS-DAO, GAQ, SSEs) consistently reported higher (less severe) scores on the EDQ relative to patients in the more severe groups (all *P* values < 0.05), providing support that the EDQ differentiates between patients with varying degrees of HSDD severity.

### Relationship between monthly and daily recall versions

The relationship between the monthly and daily recall versions of the EDQ was analyzed by calculating the Spearman’s correlation coefficient and ICC between the responses at baseline and month 3. At both baseline and month 3, the monthly and daily recall versions of EDQ scores were highly correlated (Spearman’s correlation coefficient all > 0.76; ICC: 0.64 to 0.83).

### EDQ ability to detect changes

Responders consistently reported higher (less severe) EDQ scores relative to nonresponders for all three responder criteria (all *P* values < 0.0001), providing support for the ability of the EDQ to detect change among patients known to have experienced a change in clinical status. The mean change for responders ranged between 0.7 and 1.1 for the monthly recall and between 0.6 and 0.7 for the daily recall version of the EDQ (Table 5).

**Table 5:** EDQ ability to detect changes

		Baseline to Month 6 Change	
**Monthly Recall Version**		**EDQ Total Score,** **Mean (SD)**	***P***
FSDS-DAO total score severity groups^a^	< 11 (*n* = 110)	1.1 (0.90)	< 0.0001
	> 11 (*n* = 721)	0.2 (0.63)	
GAQ desire item^b^	> 5 (*n* = 370)	0.7 (0.80)	< 0.0001
	< 5 (*n* = 459)	−0.0 (0.49)	
SSEs^c^	> 2 (*n* = 213)	0.7 (0.84)	< 0.0001
	< 2 (*n* = 618)	0.1 (0.65)	
**Daily Recall Version**		**EDQ Total Score,** **Mean (SD)**	***P***
FSDS-DAO total score severity groups^a^	< 11 (*n* = 51)	0.9 (0.74)	< 0.0001
	> 11 (*n* = 242)	0.2 (0.58)	
GAQ desire item^b^	> 5 (*n* = 141)	0.6 (0.76)	< 0.0001
	< 5 (*n* = 151)	−0.0 (0.39)	
SSEs^c^	> 2 (*n* = 89)	0.6 (0.73)	< 0.0001
	< 2 (*n* = 209)	0.1 (0.58)	

*EDQ* Elements of Desire Questionnaire, *FSDS-DAO* Female Sexual Distress Scale–Desire/Arousal/Orgasm, *GAQ* General Assessment Questionnaire, *SSE* Satisfying sexual event

^a^Defined as the number of patients with a score of ≥11 (met diagnosis for hypoactive sexual desire disorder; nonresponder) vs. < 11 (normal; responder) at month 6

^b^Defined based on the GAQ desire item at month 6, ≥5 (responder) vs. < 5 (nonresponder)

^c^Defined as the number of SSEs at month 6, < 2 (nonresponder) vs. ≥2 (responder) events

### EDQ monthly recall efficacy results

**Fig. 1:**
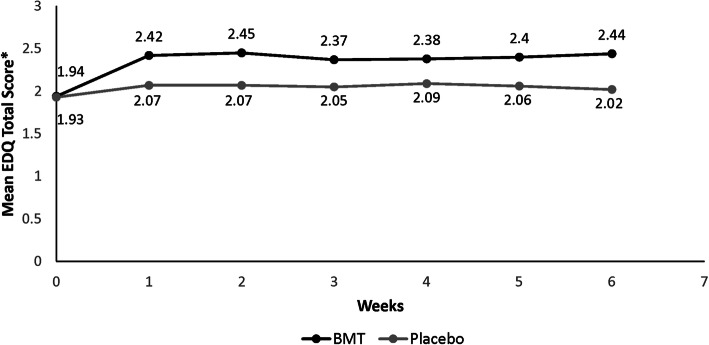
Mean EDQ total score by treatment group (BMT vs. placebo) across study visits in the integrated phase 3 RECONNECT studies – monthly recall version. BMT, bremelanotide; EDQ, Elements of Desire Questionnaire. *The mean EDQ total score for the monthly recall version was calculated as the mean of individual items (1–9) and does not represent the sum of all items. Scores range from 1 (lowest desire) to 5 (highest desire)

At baseline, the monthly EDQ mean total scores were comparable for the bremelanotide 1.75 mg and placebo treatment groups (Fig. 1)*.* After 1 month, the bremelanotide treatment group reported higher mean EDQ scores than the placebo treatment group (2.42 vs. 2.07). Higher mean scores persisted over the 6-month treatment period, with an estimated treatment difference between bremelanotide and placebo of 0.32 (CI: 0.25, 0.38; *P* < 0.0001) (Fig. 1).

### EDQ daily recall efficacy results

**Fig. 2:**
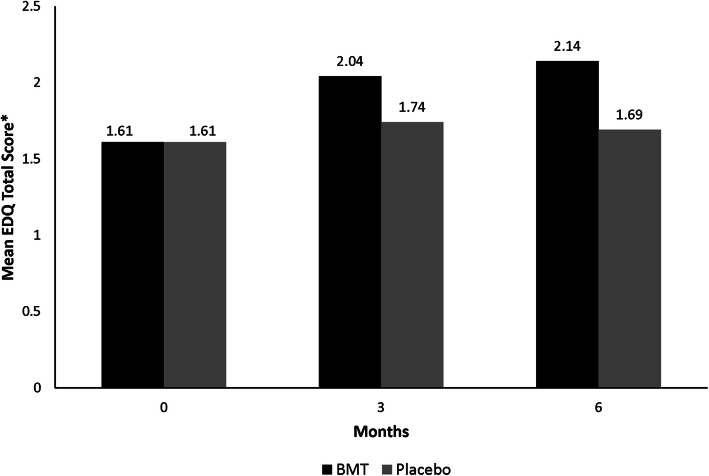
Mean EDQ total score by treatment group (BMT vs. placebo) across study visits in the integrated phase 3 RECONNECT studies – daily recall version. BMT, bremelanotide; EDQ, Elements of Desire Questionnaire. *The mean EDQ total score for the 24-h recall version was calculated as the mean of items 1, 2, 3, 4, 6, 8, and 9. Scores range from 1 (lowest desire) to 5 (highest desire). The mean score for each item was calculated for each day, and then a weekly score was calculated using these daily total scores. Data from at least 4 of the 7 days in the week were required to generate a weekly mean

At baseline, the daily EDQ mean total scores were comparable for the bremelanotide 1.75 mg and placebo treatment groups (Fig. 2). After 3 months, the bremelanotide treatment group showed higher mean EDQ scores compared with the placebo treatment group (2.04 vs. 1.74), which persisted until month 6 (2.14 vs. 1.69) (Fig. 2). At 6 months, the estimated treatment difference between bremelanotide and placebo was 0.33 (95% CI: 0.24, 0.42; *P* < 0.0001), indicating a significant improvement in desire. Consistent with the monthly EDQ results, the daily recall EDQ results showed a significant improvement for the bremelanotide treatment groups compared with the placebo treatment groups (Figs. 1 and 2).

### EDQ factor analysis

In the EFA, one- and two-factor models were extracted for the monthly recall version of EDQ. A single factor model demonstrated acceptable fit (comparative fit index = 0.978, standardized RMS residual = 0.048, and RMS error of approximation = 0.143; all item factor loadings > 0.40). A two-factor model also demonstrated acceptable fit (comparative fit index = 0.996, standardized RMS residual = 0.022, and RMS error of approximation = 0.071). The second factor included only two items (item 7 and item 8) that loaded > 0.40) and the correlation between the two factors was high (0.726), indicating that the two factors could reasonably be combined into a single factor. For the CFA, the unidimensional model fit adequately (comparative fit index = 0.990, weighted RMS residual = 1.342, and RMS error of approximation = 0.113; all item factor loadings > 0.70).

Similar results were obtained for the 24-h recall version of EDQ. In the CFA, the one-factor model demonstrated acceptable fit (comparative fit index = 0.998, standardized RMS residual = 0.040, and RMS error of approximation = 0.083). As with the monthly recall version, the two-factor model also demonstrated acceptable fit (comparative fit index = 1.000, standardized RMS residual = 0.018, and RMS error of approximation = 0.037), with high factor correlation between the first and second factors (0.733) indicating that the items can be reasonably combined into a single score. For the CFA, the unidimensional model fit adequately (comparative fit index = 0.996, weighted RMS residual = 1.271, and RMS error of approximation = 0.104; all item factor loadings > 0.75).

## Discussion

HSDD has been shown to negatively affect women’s health-related quality of life, including physical and mental health [38]. A recent study evaluating the burden of illness associated with HSDD in women demonstrated the pervasive effect of HSDD on sexual and mental health, social relationships, and overall quality of life (Simon et al., manuscript in preparation). The overall burden of HSDD was driven by impact on the relationship with their partner, mental and emotional well-being, and household and personal activities. Given the impact of HSDD on health-related quality of life and overall well-being, it is important to include PRO instruments that measure symptom severity in clinical trials to appropriately assess treatment benefit in patients with HSDD.

The EDQ is a recently developed PRO measure that evaluated the efficacy of bremelanotide versus placebo in premenopausal women with HSDD in the RECONNECT phase 3 clinical studies. The desire concepts within the EDQ are critical for HSDD diagnosis and evaluation of efficacy of HSDD therapies, as they provide additional elements to measure sexual desire and interest, including intensity of sexual desire, thoughts/fantasies about sex, and receptivity to sexual requests, which are not measured by other PRO instruments. The present study is the first comprehensive assessment of the measurement properties of the EDQ. The results provide evidence supporting the reliability and validity of the EDQ as a measure of sexual desire, and confirm that the EDQ is well suited for use as a clinical outcome assessment in clinical trials evaluating treatments for HSDD.

Factor analysis supported a unidimensional model for both the monthly and 24-h recall version of the EDQ, and confirms the factor structure in women with HSDD. Although the RMS error assessment was higher than the minimum threshold of 0.08, it should be noted that elevated values are often observed in simple models such as this, and do not weaken the conclusions [39]. The EDQ demonstrated excellent internal consistency and good evidence of test-retest reliability. The EDQ monthly scores had internal consistency (0.85 to 0.96) at all time points, as did the EDQ daily weekly score (0.91 to 0.97).

The construct validity of the EDQ was supported through moderate to large correlations between the EDQ and FSFI-D, FSEP-R, and GAQ Item 2 (convergent validity), and smaller correlations between the EDQ and more distal measures such as the FSFI-pain domain. The majority of prespecified expectations on the magnitude of correlations between the EDQ and other measures of desire and sexual function were confirmed. The known-group validity analysis demonstrates that the EDQ differentiates between the lowest and highest HSDD severity groups based on three separate anchor variables (FSDS-DAO, GAQ desire item, and number of SSEs). Collectively, these results provide support for the construct validity of the EDQ.

For the test-retest reliability results, although the ICC values did not meet an acceptable range (monthly recall = 0.63; daily recall = 0.33), the values did improve when limited to a stable population, as defined by a change of no more than ±5% in the FSDS-DAO total score (monthly recall scores = 0.68; daily recall weekly scores = 0.58). These results may indicate that there are natural daily fluctuations in patient symptoms over a daily period, and that a monthly recall version may be more appropriate. Additional research is needed to further evaluate the test-retest reliability in this patient population, as well as the natural fluctuations in desire among healthy women and women with HSDD.

Notably, there was a substantially higher rate of missing data for the daily recall version relative to the monthly recall version of the questionnaire. Patients noted inconsistency for completing the daily diary version due to the infrequency of their current sexual activity and the lack of day-to-day variations experienced with sexual desire. Given these sentiments and the high rate of missing data for the daily diary version, the monthly recall version of the EDQ may be more appropriate for a clinical trial context. The EDQ monthly recall version may provide a more meaningful method for evaluating changes in sexual desire over time. The daily questionnaire may also contaminate the results by repeated questioning [21]. Furthermore, there may be additional limitations to this psychometric analysis. The study inclusion and exclusion criteria and demographic profile of patients willing to participate in a clinical trial may impact how well the results generalize to a broader population.

Currently, the recommended primary endpoints for HSDD clinical trials focus on measures of sexual desire and interest and personal distress [40]. The FSFI-D is recommended for the assessment of sexual desire, and the FSDS-DAO Item 13 (personal distress related to low sexual desire) is recommended for the assessment of personal distress. Based on the current study, the EDQ may represent an acceptable and more comprehensive measure of sexual desire and interest for HSDD clinical trials, with FSDS-DAO Item 13 measuring personal distress.

Nevertheless, based on this initial psychometric analysis, there is evidence to support the use of the EDQ in assessment of desire concepts in patients with HSDD. The results demonstrate that the factor structure of the EDQ is confirmed in this sample of women with HSDD, and the internal consistency, reliability, construct validity, and ability to detect change are adequate for use in women with HSDD. There were moderate to large correlations between both the daily and monthly recall versions of the EDQ, and currently available tools that measure sexual desire, namely, the FSFI-D. The correlation of the EDQ monthly and daily recall versions is also noteworthy; given this correlation and inherent issues with daily data collection (i.e., missing data), the EDQ monthly recall version has demonstrable advantages. Furthermore, the EDQ should be available to the public such that clinicians can utilize it for clinical trials or daily practice. Overall, the EDQ is a novel PRO instrument that provides additional insights for evaluating potential treatments for low sexual desire, one of the well-defined hallmarks of HSDD.

## Conclusions

Psychometrically sound measures of sexual desire are necessary for HSDD clinical trials. This study found that the EDQ exhibited good reliability, validity, and sensitivity to change in evaluating sexual desire in women diagnosed with HSDD. Consistent with other previously developed measures of sexual desire and interest, the EDQ provides additional insights into a broader range of sexual desire attributes. The EDQ may represent an improved endpoint for clinical trials comparing treatments for HSDD.

## Data Availability

The dataset analyzed during the current study is available from the corresponding author on reasonable request.

## Abbreviations

BMI: Body mass index
BMT: Bremelanotide
CFA: Confirmatory factor analysis
CFI: Comparative fit index
CI: Confidence interval
DSM: Diagnostic and Statistical Manual of Mental Disorders
DSM-IV: Diagnostic and Statistical Manual of Mental Disorders, Fourth Edition
DSM-5: Diagnostic and Statistical Manual of Mental Disorders, Fifth Edition
EDQ: Elements of Desire Questionnaire
EFA: Exploratory factor analysis
FDA: US Food and Drug Administration
FSD: Female sexual dysfunction
FSDS-DAO: Female Sexual Distress Scale–Desire/Arousal/Orgasm
FSDS-R: Female Sexual Distress Scale–Revised
FSEP-R: Female Sexual Encounter Profile–Revised
FSFI: Female Sexual Function Index
FSFI-D: Female Sexual Function Index–desire domain
GAQ: General Assessment Questionnaire
GLM: General linear model
HSDD: Hypoactive sexual desire disorder
ICC: Intra-class correlations
ICH: International Conference on Harmonisation of Technical Requirements of Pharmaceuticals for Human Use
mITT: Modified intent-to-treat
OLE: Open-label extension
PRO: Patient-reported outcome
Q: Question
RMS: Root mean square
SD: Standard deviation
SSE: Satisfying sexual event
WITS-9: Women’s Inventory of Treatment Satisfaction
